# Outer Membrane Vesicles from *Neisseria Meningitidis* (Proteossome) Used for Nanostructured Zika Virus Vaccine Production

**DOI:** 10.1038/s41598-018-26508-z

**Published:** 2018-05-29

**Authors:** Paula Martins, Daisy Machado, Thais Holtz Theizen, João Paulo Oliveira Guarnieri, Bruno Gaia Bernardes, Gabriel Piccirillo Gomide, Marcus Alexandre Finzi Corat, Camilla Abbehausen, José Luiz Proença Módena, Carlos Fernando Odir Rodrigues Melo, Karen Noda Morishita, Rodrigo Ramos Catharino, Clarice Weis Arns, Marcelo Lancellotti

**Affiliations:** 1 Faculty of Pharmaceutical Sciences - FCF, University of Campinas – UNICAMP, Campinas, São Paulo, Brazil; 2 Biotechnology Laboratory, LABIOTEC, Biochemistry and Tissue Biology Department, Institute of Biology, University of Campinas – UNICAMP, Campinas, São Paulo, Brazil; 3 Multidisciplinary Center for Biological Research, University of Campinas, Campinas, São Paulo Brazil; 4 Genetic Molecular Biology and Bioagents Department, Institute of Biology, University of Campinas – UNICAMP, Campinas, São Paulo, Brazil; 5 Inorganic Department, Institute of Chemistry, University of Campinas – UNICAMP, Campinas, São Paulo, Brazil; 6 INNOVARE Biomarkers Laboratory, Faculty of Pharmaceutical Sciences - FCF, University of Campinas – UNICAMP, Campinas, São Paulo, Brazil

## Abstract

The increase of Zika virus (ZIKV) infections in Brazil in the last two years leaves a prophylactic measures on alert for this new and emerging pathogen. Concerning of our positive experience, we developed a new prototype using Neisseria meningitidis outer membrane vesicles (OMV) on ZIKV cell growth in a fusion of OMV in the envelope of virus particles. The fusion of nanoparticles resulting from outer membrane vesicles of *N*. *meningitidis* with infected C6/36 cells line were analyzed by Nano tracking analysis (NTA), zeta potential, differential light scattering (DLS), scan and scanning transmission eletronic microscopy (SEM and STEM) and high resolution mass spectometry (HRMS) for nanostructure characterization. Also, the vaccination effects were viewed by immune response in mice protocols immunization (ELISA and inflammatory chemokines) confirmed by Zika virus soroneutralization test. The results of immunizations in mice showed that antibody production had a titer greater than 1:160 as compared to unvaccinated mice. The immune response of the adjuvant and non-adjuvant formulation activated the cellular immune response TH1 and TH2. In addition, the serum neutralization was able to prevent infection of virus particles in the glial tumor cell model (M059J). This research shows efficient strategies without recombinant technology or DNA vaccines.

## Introduction

The burden of ZIKV virus infection in Brazil and Latin America induced a run to produce new strategies to combat and prevent the infection and dissemination of this important emergent virus^1,2^. The ZIKV infection in humans is usually presented as a rash-febrile illness, in association with conjunctivitis and symptoms as headache, myalgia, arthralgia and photophobia^3^. Furthermore, the infection with ZIKV is also associated with development of Guillain-Barré syndrome, a condition associated with temporary paralysis of the lower limbs^4,5^, and congenital alterations, such as microcephaly, when women are infected during pregnancy. This Zika congenital syndrome described in Brazil in 2015 is now documented in other countries in different regions of the world^6^.

ZIKV is transmited by *Aedes aegypti* and others species of *Aedes*^7^, equivalent to Dengue and Chikungunya viruses. However, others vector-independent ways of transmission were described after ZIKV introduction in Brazil^8^, including by blood transfusion, sexual intercourse and direct contact. In fact, ZIKV is detected in different body fluids such as saliva, urine and tear^9–13^. These alternative routes of viral transmission may contribute to the faster spreading of ZIKV infection in America.

The severe clinical complications associated with ZIKV infection and the continuous circulation of this virus in invertebrate vectors and animals reservoirs (leading the possibility of new outbreaks in the near future), are illustrative of the urgent need to develop a vaccine against ZIKV^14–17^.

In this scenario, the use of Outer membrane vesicles (OMV) vaccines could be a good strategy to develop an effective and cheap vaccine against ZIKV. The production of OMV is a common feature of all gram-negative bacteria, for some reason not yet fully understood^18–21^. OMVs are produced when small portions of outer membrane are projected outward and released from the bacterial cell. Soluble proteins are associated with OMVs in the periplasm and externally as adhesive material. Such nanovesicles may spread away from the cell and have different functions in the environment, mainly in a context with bacterial biofilm. OMVs are essential for pathogenesis, quorum sensing, nutrient acquisition and even in the horizontal gene transfer^22–26^. In addition, proteins associated with OMVs exhibit several biological activities^27,28^. In brief, OMVs act a delivery vesicles contributing for bacterial survival and virulence^29–31^.

These vesicles derived from pathogens have been used for development of immunogenic vaccines, mainly against the respective microorganisms from which the OMV have been obtained. In addition, OMVs vaccination based approach have been used to delivery proteins with different primary, secondary and tertiary structures. Thus, the use of OMVs, coupled with advances in understanding the molecular basis of the immune response, can lead a generation of new viral vaccines, able to induce protection against different enveloped and no-enveloped viruses^32^. In the present study, we tested the use of OMVs to generate a vaccine against ZIKV in murine model.

## Results

### Characterization of ZIKV-OMV vesicles

**Figure 1:**
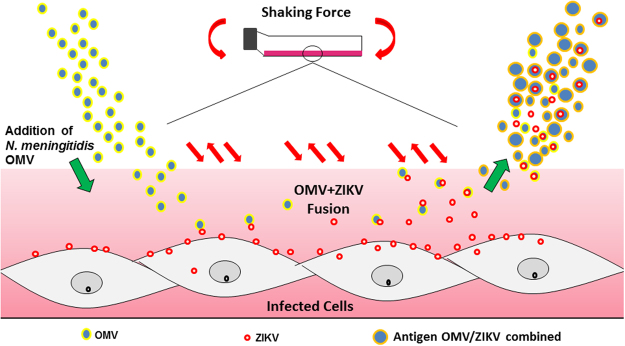
Protocol for vaccine production. Schematic representation of OMV (extracted from *N*. *meningitidis* strain C2135 in yellow and blue little circles) in fusion process of ZIKV (red circles) replicated in C6/36 cells. The agitation force promoves the fusion of OMV with vírus particle producing the OMV/ZIKV fusion particles (orange and blue circles).

In order to characterize the quality of the ZIKV-OMV vesicles, all nanovesicles generated as described in Fig. 1, were analyzed in a Zetasizer equipment. This equipment is able to measure the magnitude of the electrostatic charge and interaction among lipid particles. The parameters analyzed in Zetasizer Nano (Malvern Instruments Ltd., Grovewood Road, Malvern, United Kingdom) allow estimating the stability and causes of dispersion, flocculation and aggregation during vesicles production, which can be used for improve formulations, emulsions and suspensions during the process of vaccine production. Our results are summarized in Table 1. The ZIKV-OMV vesicles were bigger than control OMV vesicles. In addition, the ZIKV-OMV vesicles possessed polydispersity index (PDI) near of zero, revealing few interferers during the vesicles preparation. Lastly, the electric charge of the ZIKV-OMV and control-OMV vesicles was −0.429 +/−0.29 and −12 mV, respectively.

**Table 1:** Characterization by Zetasizer.

Parameter	OMV	OMV/ZIKV_fusion_
Size (nm)	192,60 ± 6,10	230,25 ± 20,85
PDI	0,513	0,572 ± 0,02
ζ Potential (mV)	−12,0 ± 1,9	−0,429 ± 0,29

Characterisation of the nanoparticles of OMV samples and OMV/ZIKV fusion.

In order to better characterize and validate these vesicles, we submitted ZIKV-OMV and control-OMV vesicles to an additional test, named NTA test (NanoSign Equipament for Nano Tracking Analysis, Malvern Instruments Ltd., Grovewood Road, Malvern, United Kingdom). In this test, the vesicles are submitted to a directional flow, where the movement speed is indirectly proportional to the size of the analyzed vesicles. The results were summarized in Table 2. As demonstrated in the Zetasizer device, ZIKV-OMV (268.9 +/− 23.5 nm) was bigger than control-OMV (192.6 +/− 6.1 nm) vesicles.

**Table 2:** Characterization by NTA of the nanoparticles of OMV samples, OMV/ZIKV fusion.

Parameter	OMV	OMV/ZIKV fusion
Size (nm)	192,60 ± 6,10	253,5 ± 5,0
D90 (nm)	268,9 ± 23,5	387,3 +/− 14,2
Particles.mL^−1^ (PPML)	1.91 ± 0,16.10^8^	1.18 ± 0,094. 10^9^
PPF	97,1 ± 8,0	60,0 ± 4,8
Centres/frame	94,9 ± 5,8	66,1 ± 6,5

According to the results obtained both by Zetasizer and the NTA, the nanoparticle of interest for the vaccine composition (OMV/ZIKV_fusion_) presents compatible size to the diameter of the OMVs described in the literature^33^. Furthermore, this size of ZIKV-OMV vesicles was compatible with vesicles used for intramuscular or subcutaneous administration^33–35^.

**Figure 2:**
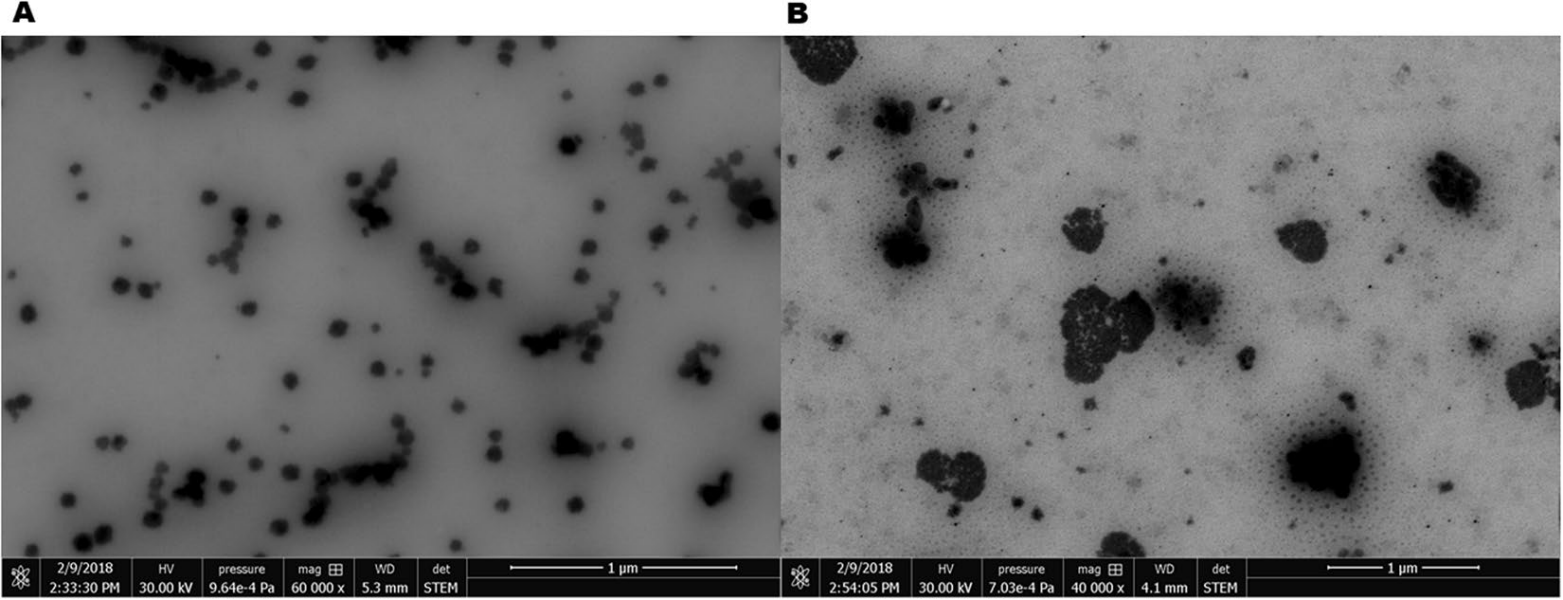
Field emission electronic microscopy. In (**A**) Scanning Transmission Electronic Microscopy - STEM the OMV nanostructure from *N*. *meningitidis*. In (**B**) STEM of OMV/ZIKVfusioned nanoparticle. The size increasing of the nanostructures showed in (**B**) when compared with the OMV in (**A**).

The STEM showed the existence of size increase in ZIKV-OMV vesicles compared with initicial OMV (Fig. 2). The nanostructures demonstrated by STEM allowed the existence of probably fusion between ZIKV and OMV (Fig. 2A,B). The size increase of the particles passed by fusion process (Fig. 2B) when compared with non fusioned particles showed in the Fig. 2A indicate a successfull process of vaccine formulation (ZIKV and OMV).

**Figure 3:**
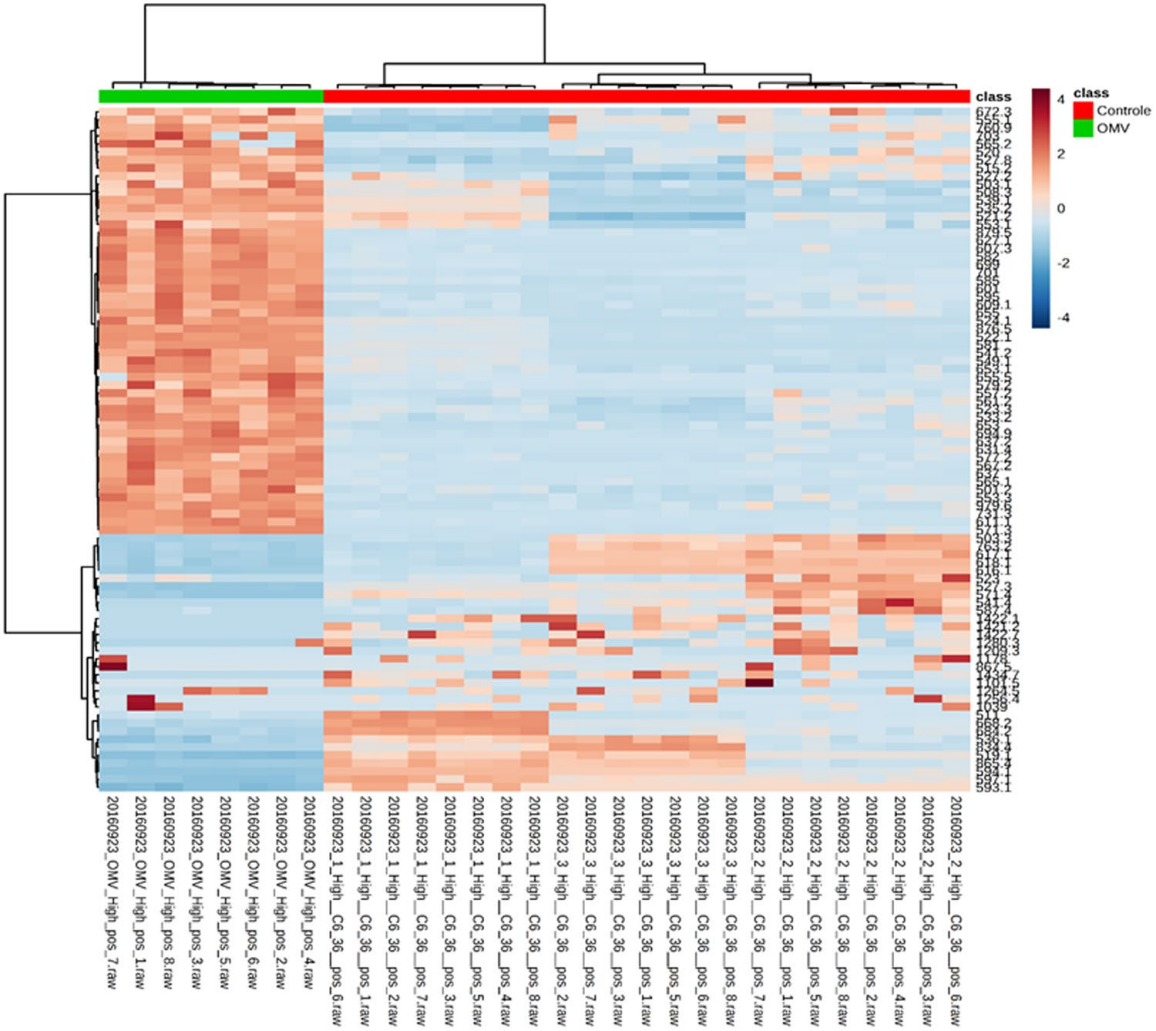
Clustering result for the 85 top features in the PLS-DA VIP scores, shown as a heat map (distance measured by Euclidean and clustering algorithm using ward.D), with a color-coded thermometer (bottom) indicating the relative presence of metabolites on each respective group.

Further the (OMV/ZIKV_fusion_) analysis by HRMS, the comparison between the OMV from *N*. *meningitidis* and those obtainef from a fusion process allowed to deteted the presence of ZIKV lipid markers demonstrating the existance of a great quantity of ZIKV epitopes in this new nanostruture or vaccine formulation. The Fig. 3 shows the existance of these ZIKV lipid markers (in red) comparing with the OMV not exposed to virus. Also, the Table 3 containg the identification of the molecules that was possible identified by HRMS specific from ZIKV.

**Table 3:** *LIPID MAPS® Lipidomics Gateway.

Experimental Mass	Teorical Mass	Error	Aduto	Molecules	ID
506,4190	506,4180	2,0	[M + Na]^+^	O-behenoylcarnitine	LMFA07070089
521,2802	521,2792	1,9	[M + H]^+^	dolichyl diphosphate	LMPR03090023
524,2973	524,2983	1,9	[M + H]^+^	PS(18:1/0:0)	LMGP03050001
537,4503	537,4513	1,9	[M + H]^+^	DG(15:1/15:1/0:0) and/or	LMGL02010327
				DG(12:0/18:2/0:0 and/or	LMGL02010337
				DG(13:0/17:2/0:0) and/or	LMGL02010358
				DG(14:1/16:1/0:0)	LMGL02010403
545,2732	545,2721	2,0	[M + Na]^+^	14-O-(β-D-glucopyranosyl)−7S	LMFA13010034
581,5128	581,5139	1,9	[M + H]^+^	DG(16:1/17:0/0:0) and/or	LMGL02010013
				DG(16:0/17:1/0:0) and/or	LMGL02010014
				DG(13:0/20:1/0:0) and/or	LMGL02010365
				DG(14:1/19:0/0:0) and/or	LMGL02010411
				DG(15:0/18:1/0:0) and/or	LMGL02010432
				DG(15:1/18:0/0:0)	LMGL02010455
593,2710	593,2721	1,9	[M + H]^+^	PI(18:4/0:0)	LMGP06050017
603,3668	603,3656	2,0	[M + H]^+^	dolichyl β-D-glucosyl phosphate	LMPR03080014
663,4946	663,4959	2,0	[M + H]^+^	PA(12:0/21:0) and/or	LMGP10010062
				PA(14:0/19:0) and/or	LMGP10010098
				PA(17:0/16:0) and/or	LMGP10010227
				PA(18:0/15:0) and/or	LMGP10010308
				PA(21:0/12:0) and/or	LMGP10010854
				PA(20:0/13:0) and/or	LMGP10010865
				PA(19:0/14:0) and/or	LMGP10010870
				PA(16:0/17:0) and/or	LMGP10010910
				PA(15:0/18:0) and/or	LMGP10010916
				PA(13:0/20:0)	LMGP10010935
667,2841	667,2854	1,9	[M + H]^+^	PI(22:6/0:0)	LMGP06050012
689,5102	689,5115	1,9	[M + Na]^+^	DG(20:3/20:4/0:0) and/or	LMGL02010197
				DG(20:2/20:5/0:0) and/or	LMGL02010198
				DG(18:3/22:4/0:0) and/or	LMGL02010223
				DG(18:2/22:5/0:0) and/or	LMGL02010224
				DG(18:1/22:6/0:0) and/or	LMGL02010225
				DG(18:4/22:3/0:0)	LMGL02010518
851,5627	851,5644	2,0	[M + H]^+^	PI(13:0/22:1) and/or	LMGP06010057
				PI(14:1/21:0) and/or	LMGP06010101
				PI(15:0/20:1) and/or	LMGP06010119
				PI(15:1/20:0) and/or	LMGP06010148
				PI(16:0/19:1) and/or	LMGP06010164
				PI(16:1/19:0) and/or	LMGP06010182
				PI(17:1/18:0) and/or	LMGP06010226
				PI(18:1/17:0)	LMGP06010295
877,5784	877,5801	1,9	[M + H]^+^	PI(15:0/22:2) and/or	LMGP06010126
				PI(15:1/22:1) and/or	LMGP06010156
				PI(17:0/20:2) and/or	LMGP06010208
				PI(17:1/20:1) and/or	LMGP06010235
				PI(17:2/20:0) and/or	LMGP06010264
				PI(18:1/19:1) and/or	LMGP06010301
				PI(18:2/19:0)	LMGP06010322

### ZIKV-OMV vesicles is immunogenic and induces a response against ZIKV in mice

**Figure 4:**
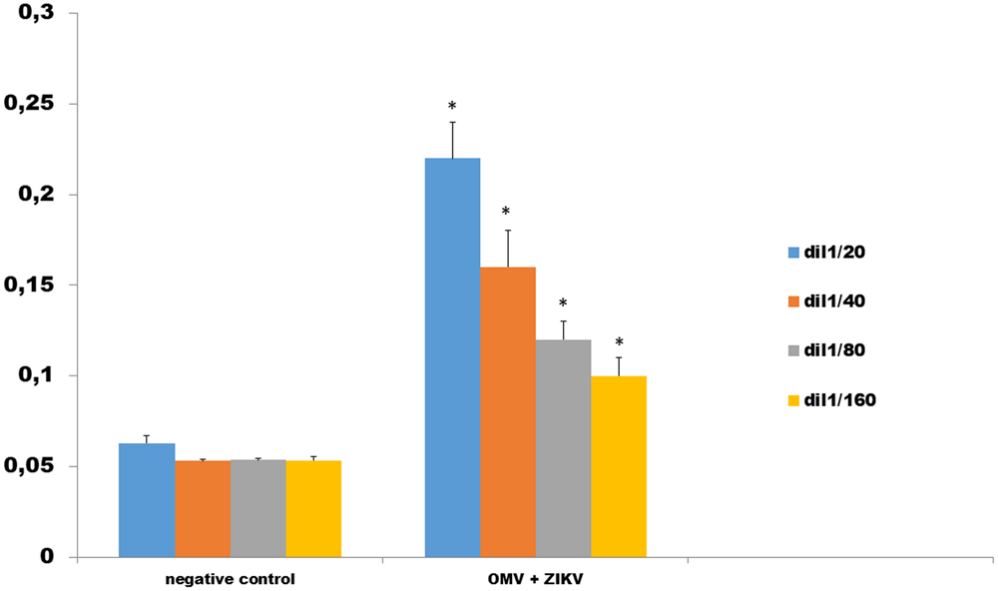
ELISA analysis of mice immunized with OMV/ZIKVfusion (group II). The antibody recognized was compared with non immunized control group (group I). The significant values were obtained until the titers 1:160.

Since we were able to generated ZIKV-OMV vesicles with good parameters of quality, we decided to analyze the potential of theses vesicles to induce an immune response against ZIKV in mice. First, we analyzed the potential of ZIKV-OMV vesicles (conjugated or not conjugated with mesoporous silica as adjuvant) to induce a production of specific IgG antibodies against ZIKV by ELISA. Both ZIKV-vaccine formulations were able to induce higher titers of ZIKV-IgG antibodies at day 14 after immunization than control-OMV vaccinated mice (Fig. 4).

**Figure 5:**
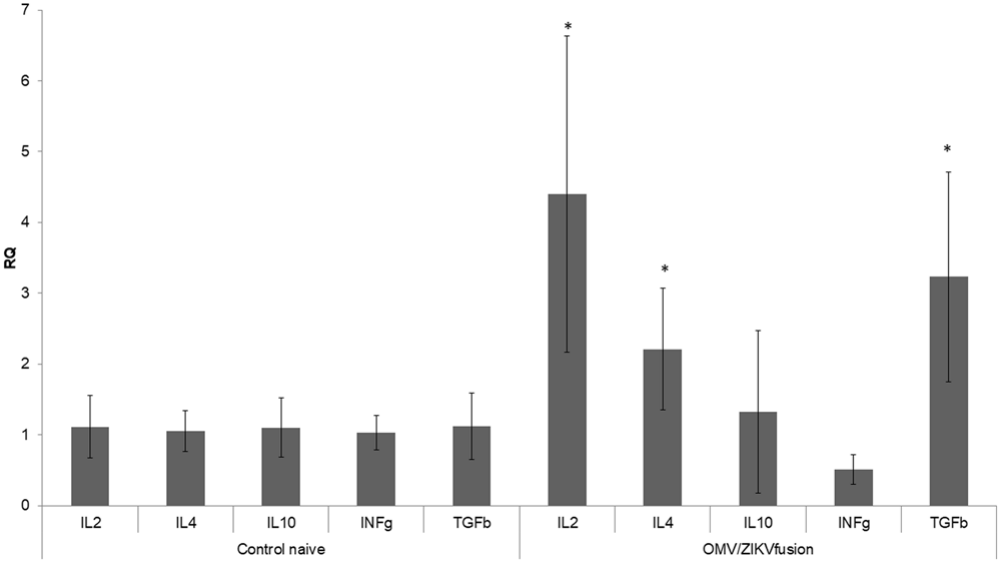
Expression of inflammatory chemokines from mice vaccined splenocytes. In this analysis were perfomed the qRTPCR using specific primers for IL2 (TH1 marker), IL4 (TH2 marker), IL10, INFγ and TGFβ (memory marker). The (*) indicate the significant imune response compared with control group not vaccined.

Despite of humoral immunological response, splenocytes of ZIKV-OMV vaccinated mice demonstrated higher levels of expression of IL-2, IL-4, and TGFβ chemokines than splenocytes from control-OMV vaccinated animals (Fig. 5). This analysis demonstrated that both TH1 and TH2 immune response are generated after ZIKV vaccination.

**Figure 6:**
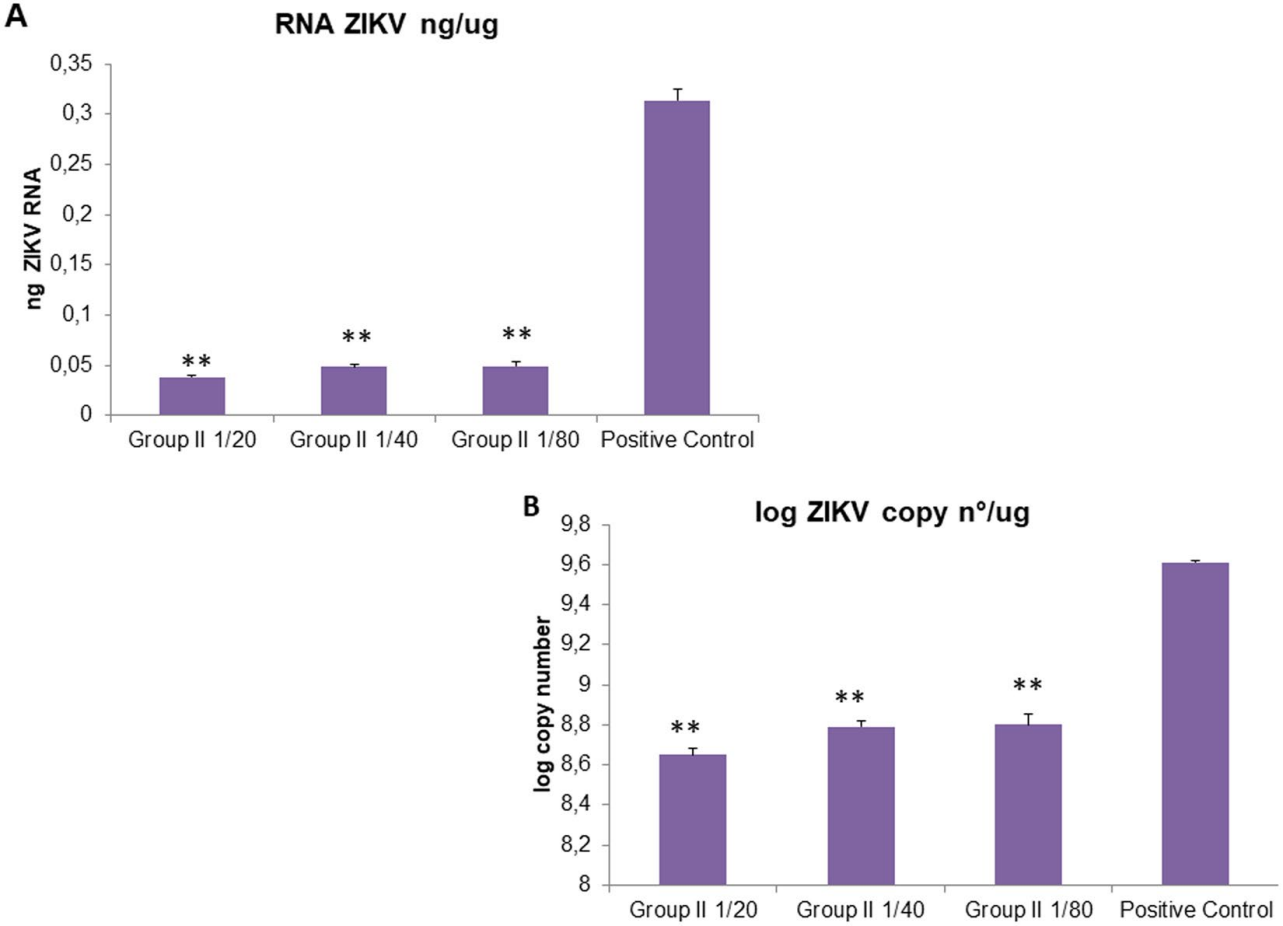
(**A**) Soroneutralization expressed in ηg/µg ZIKV particles detected in a total amount of 1 µg of RNA. The values found by qRTPCR expressed were all considered very significant with P < 0,005. (**B**) Soroneutralization expressed in log of copy number of ZIKV particles detected in a total amount of 1 µg of RNA.

In the end, the serum obtained from vaccinated mice was able to neutralize ZIKV *in vitro*, determined by a soroneutralization test in M059J cells. This experiment read by quantitative real time PCR demonstrated that serum of ZIKV-OMV vaccinated mice were able to reduce more than 1 log times the quantity of ZIKV-genome production in M059J cells in comparison with serum of control-OMV vaccinated animals (Fig. 6A,B).

The effects of the mesoporous silica adjuvance not showed an efficient role in the ZIKV vaccination (Fig. 3 Supplemental Material). The antibodies production of the group IV (adjuvated with SB16 mesoporous silica) was not induced an increase of vaccine response against ZIKV. The same results was confirmed using the soroneutralization tests, while the vaccinated group with adjuvant presence and without adjuvant not shown significant values in virus inactivation (Fig. 3 Supplemental Material).

## Discussion

Vaccines are pharmaceutical products that aim to prepare the host immune system to rapidly respond against a pathogen. The use of OMV from *Neisseria meningitidis* as methodology for vaccine production is safe because this vesicles are innocuous, biocompatible and easy to produce^33,36^. OMVs are produced by gram-negative bacteria when smaller outer membrane portions protrude outwardly and are released from the bacterial cell. Such projections are formed in the membrane regions in the absence of peptidoglycans. Soluble proteins are associated with OMVs in the periplasm and remain associated with OMVs in the external environment, such as adhesive material. The formation of OMVs can be optimized by the action of antibiotics or self-lysine (as glycosidases, amylases, peptidases, which are naturally produced by bacteria during cell division). In the present work, we choose *Neisseria meningitidis* as OMV-producer, in part because this bacterium is able to produce high amounts of OMVs that have been used for vaccine production against different pathogens^37,38^.

*Neisseria meningitidis* or meningococcus is the main cause of meningitis and sepsis throughout the world. The expressed meningococcal porin two (For A and B) outer membrane protein highly expressed in gram-negative bacteria belonging to the superfamily of porins. Porins are composed of trimeric protein of approximately 35 kDa monomers. It is known that the effects of these porins in eukaryotic cells include induction of cell activation, and immune stimulation and contribute to resistance to infection by *Neisseria* modulating survival of the host cell, and involvement in bacterial invasion into host cells^39,40^.

Also the results presented by Zetasizer, nanoparticles showed load values as expected. The OMV/ZIKV_fusion_ presented very close to zero and more positive value when compared to other nanoparticles. This result indicates greater stability of nanoparticle vaccine produced and therefore with high potential for clinical application. In addition, the results presented Pdi indicates that all samples are absent from interfering and formation of nanoparticle aggregates.

Furthermore, to ensure that the sample was inserted into the machine with precision, parameters such as PPML (particles per mL) and PPF (particles per frame) should be observed. The PPF should be in the range of 30 to 100, while the PPML must have power in the range 10^7^ and 10^9^/mL. When these values are complied with no indication that the sample is in the correct dilution, it is able to validate the NTA analysis and consequently the capacity of the real results. Also, the analysis of theses parameters were indicatif to make a dilution of each strain before their analysis (Table 2). Therefore, the characterization of the nanoparticles shows that the output was correct and efficient, to obtain the nanoparticle within the expected parameters.

Therefore, the characterization of the nanoparticles shows that the output was correct and efficient, to obtain the nanoparticle within the expected parameters. Further, the OMV/ZIKV_fusion_ characterization the imagens provides by STEM and SEM showed the existence size increasing in the OMV/ZIKV_fusion_ comparing with the simple OMV. The results provides to HRMS were decivise to characterized the ZIKV epitopes issues from pre vaccine process fusion or mix with *N*. *meningitidis* OMV’s. Indeed, the process to carry ZIKV epitopes for vaccines use were identified in a fusion condiction or a heterogenic moisture. The efficence of the antigen incorporation was verified in immune testes in ZIKV soroneutralization and antibodies recognize. In addition, the obtaintion of the moisture not identified a total fusion process for OMV and ZIKV surface particles. Thus, the use of this technology allowed the use of OMV from meningocci strain for carrier the virus antigen in a vaccine moisture.

The great impact of ZIKV in the last two years in South and Central America, with different clinical complications in adults and during pregnancy raised the need to develop new strategies for ZIKV vaccination, including those reasonably to produce in a short period of time. In this way, OMVs vaccines are good candidates, because the biotechnological processes to obtain these nanoparticles are independent of detergent extraction. In addition, OMVs allow that lipoproteins remain attached in a membrane, enhancing the immune stimulation induced by the vaccine^35,41^.

Despite of the humoral chemokines production, the vaccinated group with OMV/ZIKV fusion with and without mesoporous silica were capable to increase the IL04 (TH2 marker cytokine), IL02 (TH1 marker cytokine) and a significant TGFβ expression in groups II and III. The increasing of TGFβ production has been corroborates with the works concerning the immune memory acquired. Also, the convergent TH1 and TH2 immune response indicated a satisfactory immunization process. This process is due the presence of bacterial antigen (from OMV extracted of *N*. *meningitidis*) and virus epitopes presents in OMV/ZIKV_fusion_. The combined nanoparticle issue for this procedure carry both epitopes – bacterial and virus surface antigens.

Further, our results showed the reduction of the virus particles by soroneutralization. The serum from mice immunized with conjugated OMV/ZIKV_fusion_ (group II) and OMV/ZIKV_fusion_ SBa16 (group III) were efficient to decrease virus RNA copy number *in vitro*. The success of antibodies production and the soroneutralization capacity indicated an efficient OMV vaccine formulation for virus described in this work.

## Conclusion

This may be an important economic indicative to developing countries as Brazil, touched by ZIKV infections and microcephaly syndrome recently. Since the epidemic of infection ZIKV has caused serious pathological conditions for children and adults, prevention is the best strategy. The development of vaccine for this pathogen can then provide an improvement in public health. The transmission ZIKV is not only linked to mosquitoes of the *Aedes* species. Studies had described transmission by sex and blood transfusion, as well, and the presence of the virus in body fluids as a potential source of transmission^42,43^. This vaccine formulation is an important and efficient propose to likely low cost production and vigorous prophylactic strategy. Also, this a new approach in vaccine design without the molecular biology use for a heterologus vaccine (two different infectious diseases in this – meningitis and Zika virus), using nanostructures in the obtation process of main antigens for vaccine use. This method based in mechanical forces use should facilitate the scale-up process and decrease the cost of vaccine production. Future studies for an specific quality control of this vaccine obtantion process, its scale-up process and specific improvements in the obtaining the antigens by this new process. Also, other OMV issue from *Haemophilus influenzae* and *Neisseria* genus are target of our new studies and the use of new adjuvants nanostructured as mesoporous silica and grafen are also included into our research subjets.

## Material and Methods

### Cell, bacteria and ZIKV strains

*N*. *meningitidis* (C2135); *Aedes albopictus* (C6/36) and glial cells (M059J) used in this study were obtained from (INCQS – FIOCRUZ – National Institute for Quality Control - Oswaldo Cruz Fundation, Rio de Janeiro, RJ and Cell Bank). Brazilian ZIVK strain (BeH823339, GenBank KU729217) was provided by Professor Doctor Edison Durigon (Biomedical Sciences Institute, University of São Paulo). This viral strain was isolated by the Evandro Chagas Institute, Pará State, Brazil from a Ceará’s State (Brazil) patient in 2015.

*N*. *meningitidis* was grown at 37 °C under 5% of CO_2_ in agar GCB (Difco). C6/36 cells were cultured in Leibovitz medium, supplemented with 10% of fetal bovine serum and 1% of antibiotics and L-glutamine (2 mM). This cell was incubated at room temperature. The M059J cells line were cultivated in RPMI1640 medium supplemented with 10% of fetal bovine serum and 1% of antibiotics (levofloxacin 1 µg/mL, tetracyclin 1 µg/mL, erythromycin 3 µg/mL in hydro alcoholic 50% solution). ZIKV was replicated in C6/36 or M059J with 70% of confluence until 75% of CPE (cytopathic effect). The ZIKV aliquots of 1 mL were stored at −86 °C.

### OMVs Extraction and ZIKV OMV conjugated vaccine

OMV extraction was performed using a method of extraction by ultrafiltration following the descriptions of Alves *et al*. 2013. Filters containing OMVs were placed in 0.9% saline and stored in an oven for 20 minutes to release vesicle membranes from filter. The samples were stored at −80 °C. The vaccine preparation was carried out fusing ZIKV that were released from C6\36 cells. For this, different concentrations of OMV from *N*. *meningitidis* were added to C6/36 infected cells during different time points, in order to obtain the best vaccine preparation. The supernatants containing the OMV vesicles fused with ZIKV were collected, inactivated (56 °C for 1 hour) and then lyophilized. A brief view of the strategy used for OMV-ZIKV vesicles is demonstrated in the Fig. 1. The vaccine preparations were analyzed by Zetasizer Nano and Nano Tracking Analysis (both Malvern Instruments Ltd., Grovewood Road, Malvern, United Kingdom), Scanning Transmission Electronic Microscopy - TEM, Scan Eletronic Microscopy – SEM (Fig. 2) and High Resolution Mass Spectometry - HRMS (Fig. 3).

### Electronic microscopy – SEM and STEM

The images of the ZIKV-OMV vesicles were obtained by field emission scanning transmission electron microscopy (STEM). For STEM images, diluted aqueous suspensions were placed in a gold grid supported on carbon film, dried at room temperature. The images were acquired in a FEI scanning electron microscope, model Quanta (FEI/Thermo Scientific). Bright field STEM images were acquired at an acceleration voltage of 30 kV.

### HRMS analysis

For sample preparation, a amount of lyophilized OMV was dissolved in 1 mL of methanol and homogenized under vortex for 50 s. The obtained solution was centrifuged for 15 min under 10.000 rpm. Fifty microliters of the supernatant was then collected and diluted in 450 μL of methanol. The resulting solution was analyzed in the positive ion modes after the addition of 0.1% of formic acid. In HRMS analyses all samples were directly infused into an ESI-LTQ-XL Orbitrap Discovery (Thermo Scientific, San Jose, CA, USA) with a nominal resolution of 30,000 (FWHM). Data were acquired in the survey scan mode, according to the following parameters: flow rate at 10 μL.min^−1^, capillary temperature at 280 °C, spray current at 5 kV, and sheath gas at 5 arbitrary units. The analysis were perfomed in quintuplicate. The utilized mass range for analysis was 500–1,100 m/z. The structural proposed was performed after careful OMV spectrum analysis and interpretation of lipids data bank for molecular characterization as performed in Melo^44^ *et al*. The databases HMDB version 3.6 (Human Metabolome database—www.hmdb.ca), METLIN (Scripps Center for Metabolomics, La Jolla, CA), as well as Lipid MAPS online database (University of California, San Diego, CA—www.lipidmaps.org) were consulted. Mass accuracy was the method of choice for database research, with a maximum adopted mass error of 2 ppm.

### Ethics Statement

The mice were acquired from “Centro Multidisciplinar para Investigação Biológica na Área de Ciência em Animais de Laboratório – CEMIB/UNICAMP” (http://www.cemib.unicamp.br). The protocol for animal practice was approved in accordance with relevant guidelines and regulations. “Comissão de Ética e Uso de Animais – CEUA – UNICAMP” Comission of Ethics in animal use protocol number: 4350-1 (http://www.ib.unicamp.br/comissoes/ceua)”, the infant rats were inoculated by bacteria as described above and before the euthanasic procedure were anesthesiated by ketamine/xylasin use (50 mg/kg and 5 mg/kg, respectively).

### Mouse experiments: vaccination

Non-isogenic Swiss mice were purchased from CEMIB. All mice were bred in a specific-pathogen-free facility at IB-UNICAMP. Single dose of subcutaneous vaccination were performed in groups of five animals by injection in the back. In this study, we analyzed three different vaccine formulations: **1**. Control: only with OMV without ZIKV; **2**. OMV-ZIKV without adjuvant; **3**. OMV-ZIKV with SBa16 as adjuvant. The animals were maintained for 14 days.

### Measurement of anti-ZIKV response on vaccinated mice: specific antibodies

To determine if the vaccine formulations were able to induce a protective response against ZIKV, vaccinated mice were euthanized at 14 days after vaccination by deep anesthesia. Total bloods of all animals were collected by heart puncture, while spleen were harvested, weighed, and used for spleenocyte isolation. The presence of ZIKV specific antibodies in the serum was evaluated by ELISA assay, using ZIKV coating plates containing 500 plate forming units (pfu) for each well. As secondary antibody we used an anti–lgG mouse Whole Molecule (H + L) conjugated with peroxidase. The specific antibody response was performed as described by Virginio^45^ *et al*. 2017. The titer of each serum were perfomed in serial dilution factor 1/2 with initial dilution of 1/20 and final dilution 1/1280.

### Measurement of anti-ZIKV response on vaccinated mice: presence of neutralizing antibody against ZIKV

To determine if the vaccine preparations were able to induce the production of neutralizing antibodies, serum from all animals group were incubated with 1000 pfu of ZIKV and used to infect M059J glial cells and Vero cells. Positive control consisted of 1000 pfu of ZIKV without serum. The viral replication in these cells was determined by quantitative real time PCR after 24 h post infection. ZIKV RNA levels in these cells were determined after RNA extraction by TRIZOL using an absolute and relative quantification by Sybr one-step reverse transcriptase PCR. This reaction were adapted from Lanciotti *et al*.^11^ and the RNA copy number was obtained using the equation described by Jung *et al*.^46^.

$${\rm{Copy}}\,{\rm{number}}=(\text{conc}.{\rm{\eta }}{\rm{g}}/\text{mL}\times \mathrm{6,022}\cdot {10}^{23})/(\text{fragment}\,{\rm{length}}\times 1{.10}^{9}\times 609).$$

During relative quantification, the amplification of the glyceraldehyde- 3-phosphate dehydrogenase (GAPDH) gene (IdT catalog no. Mm.PT.39a.1) was used as a control for normalization. Briefly, all reactions were performed using 300 ng of RNA with the following cycling algorithm: 48 °C for 30 min, 95 °C for 10 min, and 45 cycles of 95 °C for 15 s and 60 °C for 1 min.

### Measurement of anti-ZIKV response on vaccinated mice: expression of inflammatory chemokines

To measure the chemokine production induced by the vaccine formulations, spleenocytes were obtained from spleen-explants grown in RPMI1640 supplemented with calf fetal sera at 10%. The cells were incubated at 37 °C in humidity and 5% carbonic dioxide controlled atmosphere. The RNA of these cells was obtained by TRIZOL and the expression of IL1β, IL2, IL4, INFγ, TNFα and TGFβ were determined by relative quantification using Sybr one-step reverse transcriptase PCR after normalization with GAPDH. The reactions conditions flowed what was described above.

### Statistical analysis

The experiments were performed in triplicate and the results are presented as mean and standard deviation. For statistical analysis, the results were compared using 01-way analysis of variance (ANOVA), with Tukey as post–test. P value of 0.05 indicated statistically significant difference. The mass spectrometric data were treated as described in Melo *et al*.^44^. Summary Partial Least Squares Discriminant Analysis (PLS-DA) was used as the method of choice to assess association between groups. The statistical significance of the PLS-DA model was assessed by two permutation tests: precision preview during modeling and separation distance; 2000 permutations were used in both tests (p < 0.05). The selection of characteristic features for OMV/ZIKA was carried out considering the impact that each feature had in the analysis through VIP (Variable Importance in Projection) scores using as cutoff threshold VIP score greater than 1.5. The heat map of the VIPs was built using the Pearson’s distance measurement and Ward’s clustering algorithm. All analyses involving PLS-DA and VIP scores were carried out using the online software MetaboAnalyst 3.0^47^.

## Electronic supplementary material

Supplementary Information
