# Supplementary material for: Outer Membrane Vesicles from Neisseria Meningitidis (Proteossome) Used for Nanostructured Zika Virus Vaccine Production
Source: Sci Rep. 2018 May 29;8:8290. doi: 10.1038/s41598-018-26508-z (PMC5974080; doi:10.1038/s41598-018-26508-z)
Supplement: Supplementary file 1 — Supplementary Information [file 41598_2018_26508_MOESM1_ESM.pdf]

OUTER MEMBRANE VESICLES FROM *NEISSERIA MENINGITIDIS* (PROTEOSSOME) USED FOR NANOSTRUCTURED ZIKA VIRUS VACCINE PRODUCTION

Paula Martins[1], Daisy Machado[2], Thais Holtz Theizen[2], João Paulo de Oliveira Guarnieri[1], Bruno Gaia Bernardes[1], Gabriel Piccirillo Gomide[1], Marcus Alexandre Finzi Corat[3], Camila Abbehausen [5], José Luiz Proença Módena[4], Carlos Fernando Odir Rodrigues Melo[6], Karen Noda Morishita[6], Rodrigo Ramos Catharino[6], Clarice Weis Arns[4], Marcelo Lancellotti[1,2]*

1 - Faculty of Pharmaceutical Sciences - FCF, University of Campinas – UNICAMP.

2 - Biotechnology Laboratory, LABIOTEC, Biochemistry and Tissue Biology Department, Institute of Biology, University of Campinas – UNICAMP.

3 - Multidisciplinary Center for Biological Research, University of Campinas, São Paulo, Brazil.

4 - Genetic Molecular Biology and Bioagents Department, Institute of Biology, University of Campinas – UNICAMP.

5 – Inorganic Department, Institute of Chemistry, University of Campinas – UNICAMP.

6 - INNOVARE Biomarkers Laboratory, Faculty of Pharmaceutical Sciences - FCF, University of Campinas – UNICAMP.

*Corresponding author: Prof. Dr. Marcelo Lancellotti. Rua Cândido Portinari, 200, Cidade Universitária Zeferino Vaz, Faculty of Pharmaceutical Sciences, University of Campinas, Campinas, São Paulo, Brazil, CEP: 13083-871 e-mail: marcelo.lancellotti@fcf.unicamp.br.

SUPPLEMENTARY INFO

Supplementary material for DLS analysis.

Parameter	OMV	OMV/$ZIKV_{fusion}$
Size (nm)	192,60/175,4/178,9	215,00/ 245/230,25
PDI	0,513/0,567/0,493	0,590/0,555/0,572
ζ Potential (mV)	-12,0/-15,3/-11,5	-0,429/-0,702/ -0,282

Figure 1

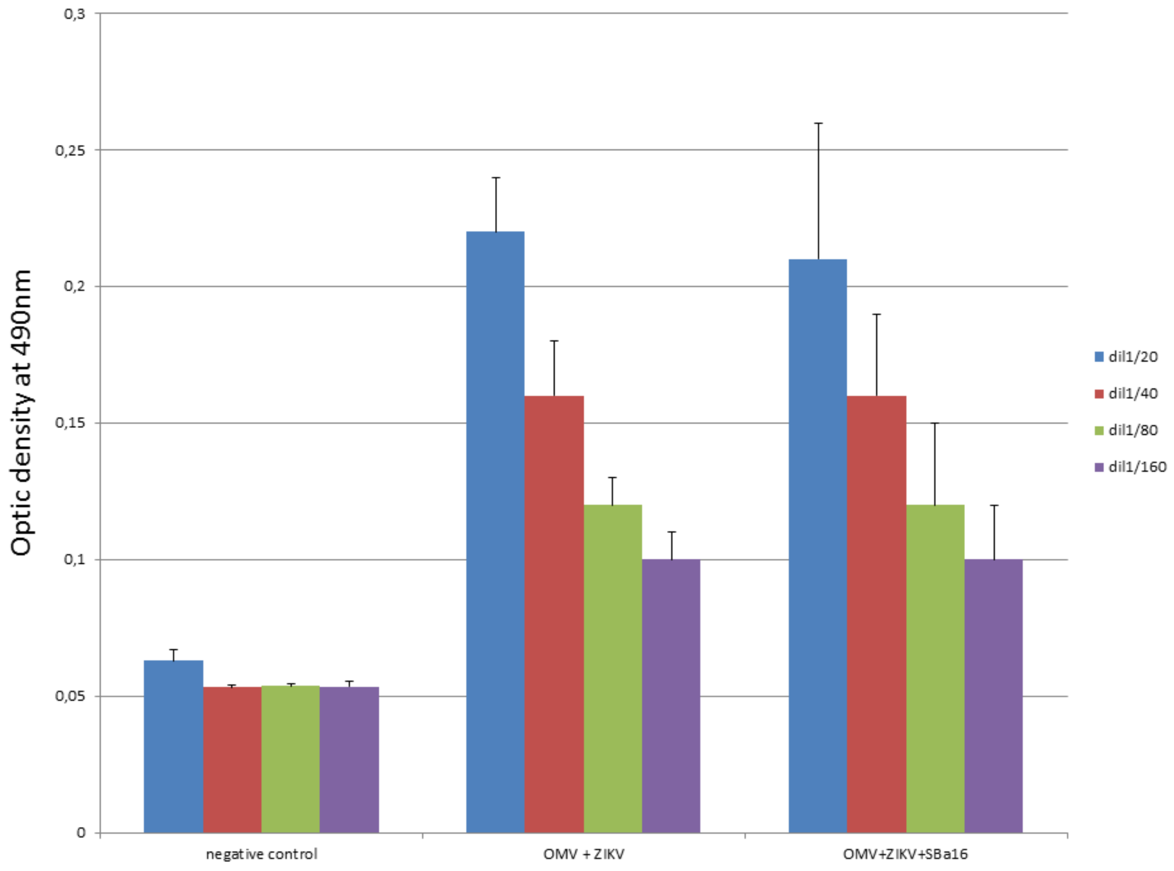

ELISA analysis of mice immunized with OMV/ZIKVfusion (group II) and OMV/ZIKV fusion + SBa16 (group III). The antibody recognized was compared with non immunized control group (group I). The significant values were obtained until the titers 1:160 in both groups (II and III).

Figure 2

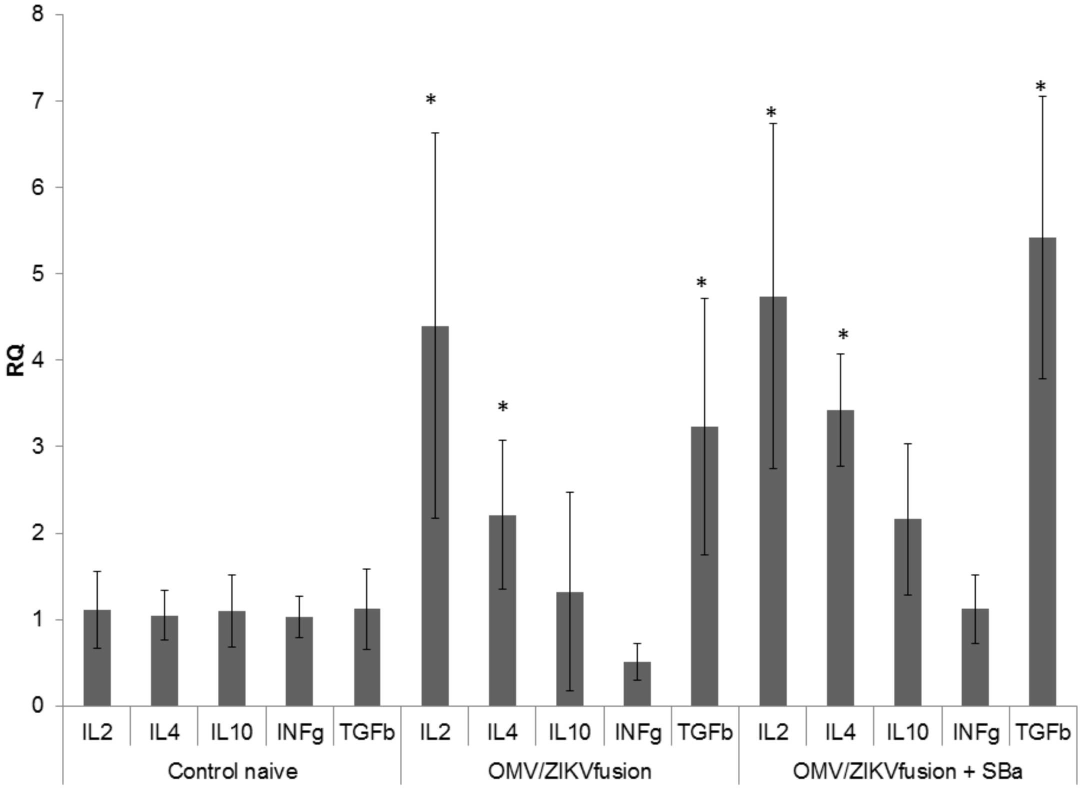

Expression of inflammatory chemokines from mice vaccined splenocytes. In this analysis were perfomed the qRTPCR using specific primers for IL2 (TH1 marker), IL4(TH2 marker), IL10, INFγ and TGFβ (memory marker). The (*) indicate the significant imune response compared with control group not vaccined.

Figure 3

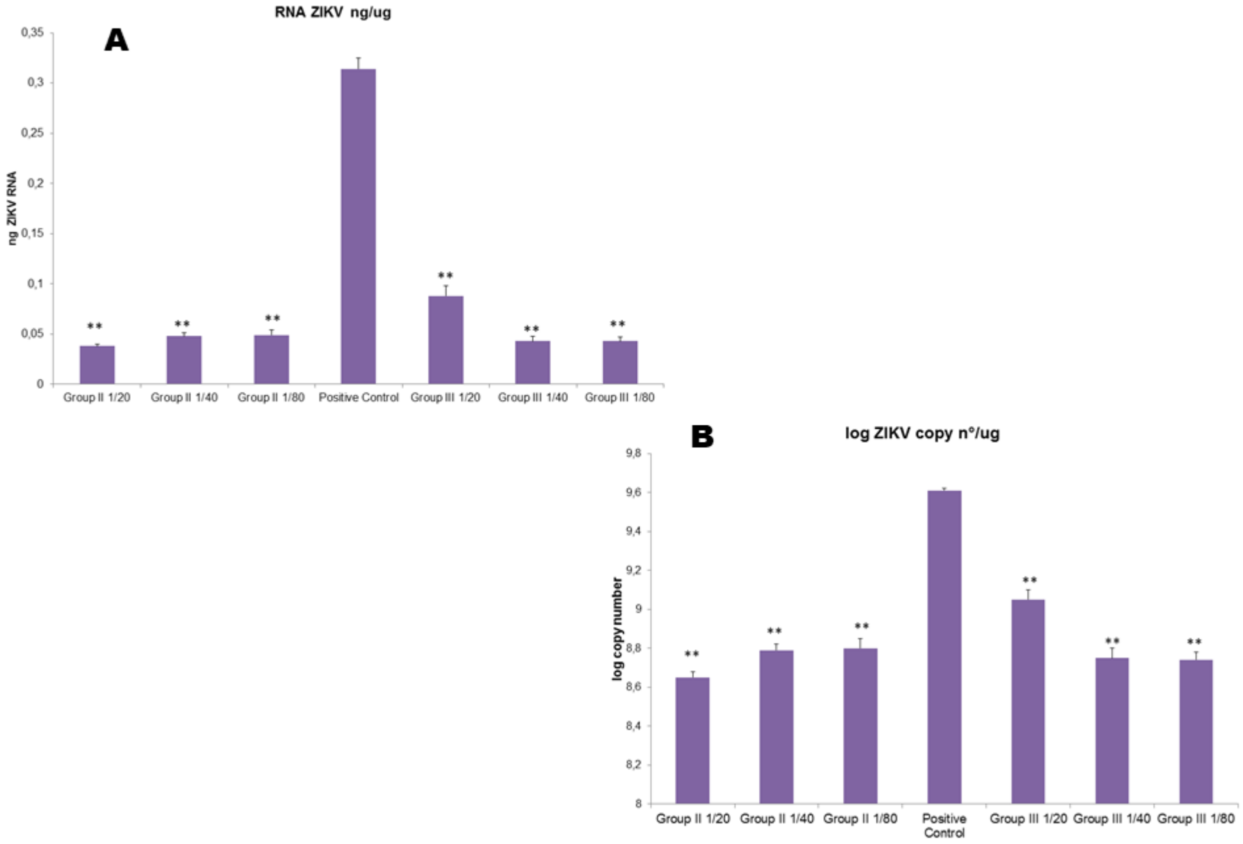

(A) Soroneutralization expressed in ηg/µg ZIKV particles detected in a total amount of 1µg of RNA. The values found by qRTPCR expressed were all considered very significant with P<0,005. (**B)** Soroneutralization expressed in log of copy number of ZIKV particles detected in a total amount of 1µg of RNA. The values found by qRTPCR expressed were all considered very significant with P<0,005.

Nta Omv c2135 2016-06-03 11-51-23

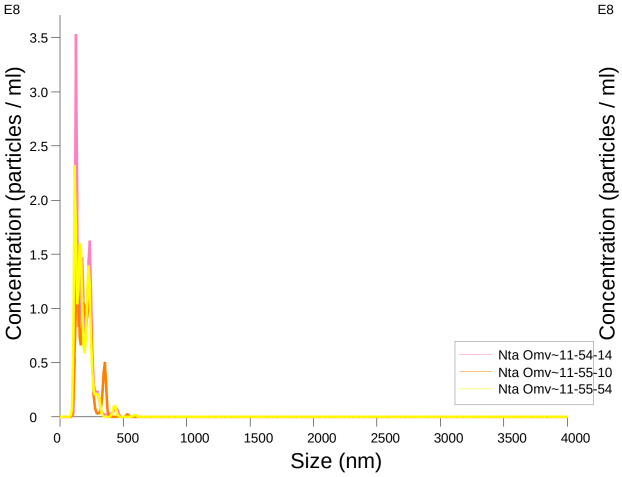

FTLA Concentration / Size graph for Experiment:
Nta Omv c2135 2016-06-03 11-51-23

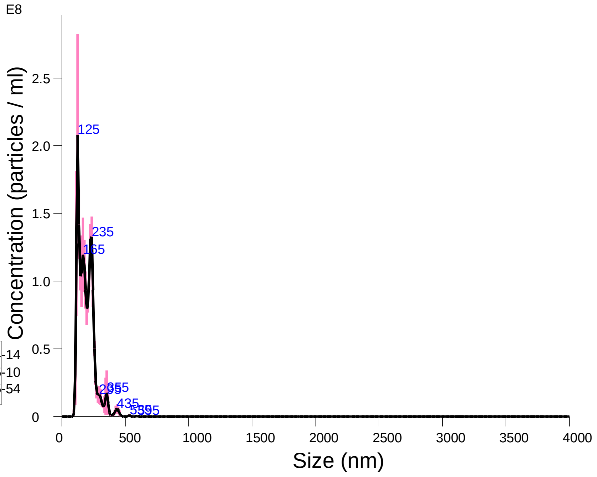

Averaged FTLA Concentration / Size for Experiment:
Nta Omv c2135 2016-06-03 11-51-23
Error bars indicate + / -1 standard error of the mean

Included Files

Nta Omv c2135 2016-06-03 11-54-14
Nta Omv c2135 2016-06-03 11-55-10
Nta Omv c2135 2016-06-03 11-55-54

Details

NTA Version:	NTA 3.1 Build 3.1.54
Script Used:	SOP Standard Measurement 11-51-13AM 03J~
Time Captured:	11:51:23 03/06/2016
Operator:	Paula
Pre-treatment:	
Sample Name:	Nta Omv c2135
Diluent:	Agua
Remarks:	

Capture Settings

Camera Type:	sCMOS
Camera Level:	12
Slider Shutter:	1200
Slider Gain:	146
FPS	18.7
Number of Frames:	562
Temperature:	24.1ºC
Viscosity:	(Water) 0.9 cP
Dilution factor:	Dilution not recorded

Analysis Settings

Detect Threshold:	12
Blur Size:	Auto
Max Jump Distance:	Auto: 10.8 - 11.6 pix

Results

Stats: Merged Data

Mean:	191.7 nm
Mode:	125.5 nm
SD:	66.3 nm
D10:	111.4 nm
D50:	170.3 nm
D90:	251.1 nm

Stats: Mean +/- Standard Error

Mean:	192.6 +/- 6.1 nm
Mode:	126.3 +/- 5.4 nm
SD:	66.0 +/- 1.6 nm
D10:	112.3 +/- 3.9 nm
D50:	170.8 +/- 8.0 nm
D90:	268.9 +/- 23.5 nm
Concentration:	1.91e+009 +/- 1.58e+008 particles/ml
	97.1 +/- 8.0 particles/frame
	94.9 +/- 5.8 centres/frame

Nta Omv c2135 2016-06-03 11-51-23

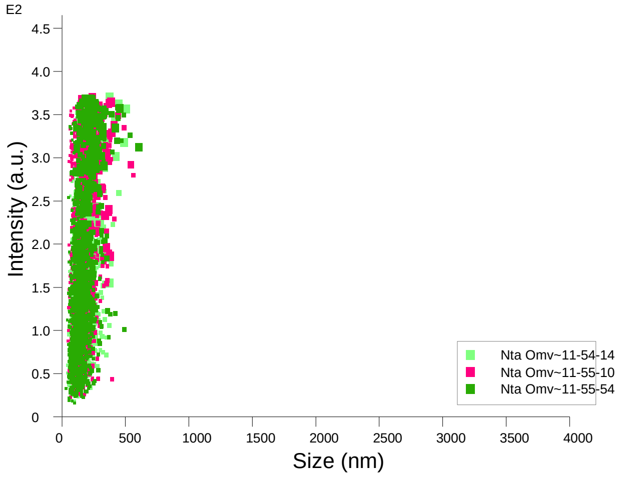

Intensity / Size graph for Experiment:
Nta Omv c2135 2016-06-03 11-51-23

Script Used: (Full Text):

SOP Standard Measurement 11-51-13AM 03Jun2016.txt

ZIKA + OMV + CELULA MOSQUITO 2 2016-07-01 14-06-44

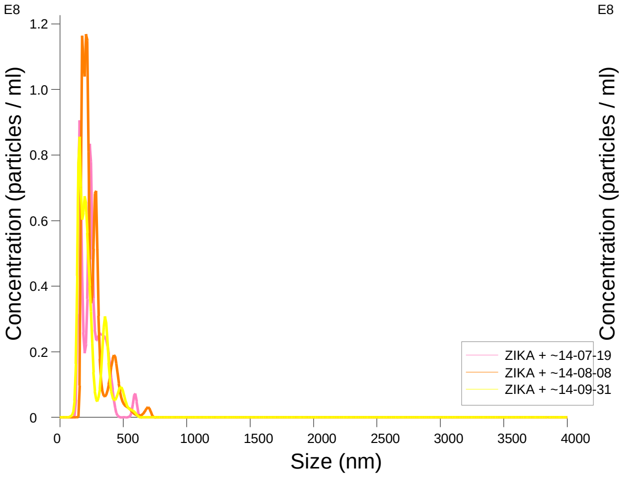

FTLA Concentration / Size graph for Experiment:
ZIKA + OMV + CELULA MOSQUITO 2 2016-07-01 14-06-44

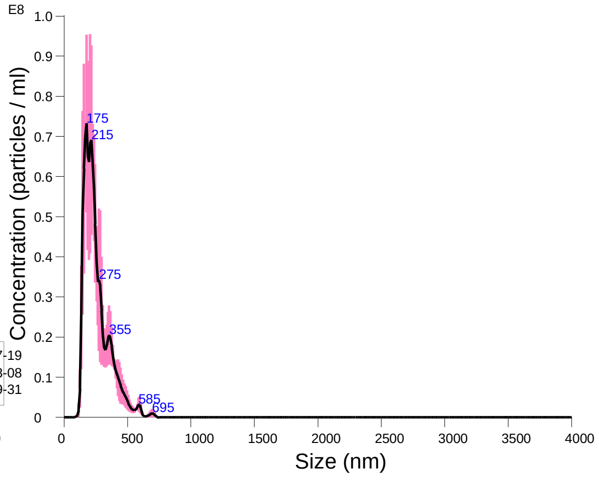

Averaged FTLA Concentration / Size for Experiment:
ZIKA + OMV + CELULA MOSQUITO 2 2016-07-01 14-06-44
Error bars indicate + / -1 standard error of the mean

Included Files

ZIKA + OMV + CELULA MOSQUITO 2 2016-07-01 14-07-19
ZIKA + OMV + CELULA MOSQUITO 2 2016-07-01 14-08-08
ZIKA + OMV + CELULA MOSQUITO 2 2016-07-01 14-09-31

Details

NTA Version:	NTA 3.1 Build 3.1.54
Script Used:	SOP Standard Measurement 12-59-22PM 01J~
Time Captured:	14:06:44 01/07/2016
Operator:	PAULA
Pre-treatment:	
Sample Name:	ZIKA + OMV + CELULA MOSQUITO 2
Diluent:	Agua
Remarks:	

Capture Settings

Camera Type:	sCMOS
Camera Level:	13
Slider Shutter:	1232
Slider Gain:	219
FPS	18.3
Number of Frames:	547
Temperature:	24.6 - 25.3 °C
Viscosity:	(Water) 0.883 - 0.897 cP
Dilution factor:	Dilution not recorded

Analysis Settings

Detect Threshold:	8
Blur Size:	Auto
Max Jump Distance:	Auto: 8.7 - 10.8 pix

Results

Stats: Merged Data

Mean:	254.2 nm
Mode:	173.1 nm
SD:	100.8 nm
D10:	145.2 nm
D50:	215.4 nm
D90:	385.6 nm

Stats: Mean +/- Standard Error

Mean:	253.5 +/- 5.0 nm
Mode:	172.1 +/- 18.3 nm
SD:	100.4 +/- 3.6 nm
D10:	146.6 +/- 9.3 nm
D50:	213.8 +/- 8.9 nm
D90:	387.3 +/- 14.2 nm
Concentration:	1.18e+009 +/- 9.39e+007 particles/ml
	60.0 +/- 4.8 particles/frame
	66.1 +/- 6.5 centres/frame

ZIKA + OMV + CELULA MOSQUITO 2 2016-07-01 14-06-44

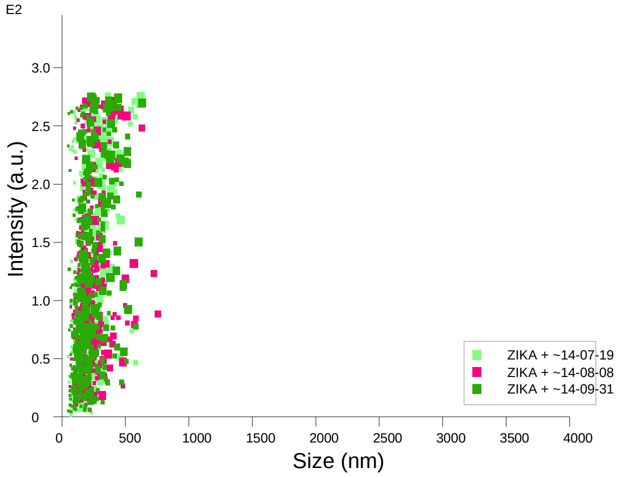

Intensity / Size graph for Experiment:
ZIKA + OMV + CELULA MOSQUITO 2 2016-07-01 14-06-44

Script Used: (Full Text):

SOP Standard Measurement 12-59-22PM 01Jul2016.txt
